# Dr. Paris's Pharmacologia, 4th Edition Enlarged

**Published:** 1820-12-01

**Authors:** 


### XIII.

*Pharmacologia; or the History of Medicinal Substances, with a view to establish the Art of prescribing and of composing Extemporaneous Formulæ, upon fixed and scientific Principles; illustrated by Formulæ, in which, the Intention of each Element is designated by Key Letters.* By John Ayrton Paris, M.D. F.L.S. M.R.I. Fellow of the Royal College of Physicians, &c. &c. &c. *Fourth Edition*, much enlarged. One Vol. Octavo, pp. 580. London, 1820.

Our review of the *third* edition of this work was scarcely out of the press, when the edition itself was out of print. Now, while we admit that some medical works of real merit are passed over by the public, without notice, and suffered to remain in obscurity; yet we believe that there is not a single instance on record, at least in modern times, of a strictly medical work going through three or four editions, and thus receiving public approbation, without possessing intrinsic merits. If the above position be correct, (and we believe that no unbiassed observer will question its correctness) Dr. Paris's work is stamped with the authentic and irrevocable seal of merit. We have, therefore, but to announce this new, and greatly enlarged, as well as improved edition; and allow things to take their natural and regular course.

This edition is dedicated in a tone of manly friendship and fine feeling, to Dr. Maton, who is justly represented as earnest "in upholding the dignity, and encouraging the legitimate exercise of our profession,"—a physician eminently enlightened in natural history, and, consequently, capable of justly appreciating the importance of its applications to medicine; while the extensive practice which his talents and urbanity so deservedly command in this metropolis, must, long since, have taught him the full extent of that empiricism, which it is Dr. Paris's endeavour to expose, and that ignorance which it is his object to enlighten.

Would to God that all the members of a liberal and enlightened profession thus acted and felt towards each other! It is in vain for us to expect the perfect esteem of the public, till we determine to respect ourselves, and act honourably towards others.

It was our intention to have noticed some of the numerous additions and improvements in this edition, but we find that a long article would give but a very imperfect view of them, and the limits of this Number of the Journal are already far exceeded. The rapidity with which the work is now flowing through the various ramifications of the profession, renders all delineation of its contents a work of supererogation.

——— quo non aliud velocius ullum
Mobilitate viget, viresque acquirit eundo.

